# Polymorphisms of the μ‐opioid receptor gene influence cerebral pain processing in fibromyalgia

**DOI:** 10.1002/ejp.1680

**Published:** 2020-11-02

**Authors:** Isabel Ellerbrock, Angelica Sandström, Jeanette Tour, Diana Kadetoff, Martin Schalling, Karin B. Jensen, Eva Kosek

**Affiliations:** ^1^ Department of Clinical Neuroscience Karolinska Institutet Stockholm Sweden; ^2^ Department of Neuroradiology Karolinska University Hospital Stockholm Sweden; ^3^ Stockholm Spine Center Löwenströmska Hospital Upplands Väsby Sweden; ^4^ Department of Molecular Medicine and Surgery Karolinska Institutet Stockholm Sweden; ^5^ Center for Molecular Medicine Karolinska University Hospital Stockholm Sweden

## Abstract

**Background:**

Dysregulation of the μ‐opioid receptor has been reported in fibromyalgia (FM) and was linked to pain severity. Here, we investigated the effect of the functional genetic polymorphism of the μ‐opioid receptor gene (OPRM1) (*rs1799971*) on symptom severity, pain sensitivity and cerebral pain processing in FM subjects and healthy controls (HC).

**Methods:**

Symptom severity and pressure pain sensitivity was assessed in FM subjects (*n* = 70) and HC (*n* = 35). Cerebral pain‐related activation was assessed by functional magnetic resonance imaging during individually calibrated painful pressure stimuli.

**Results:**

Fibromyalgia subjects were more pain sensitive but no significant differences in pain sensitivity or pain ratings were observed between OPRM1 genotypes. A significant difference was found in cerebral pain processing, with carriers of at least one G‐allele showing increased activation in posterior cingulate cortex (PCC) extending to precentral gyrus, compared to AA homozygotes. This effect was significant in FM subjects but not in healthy participants, however, between‐group comparisons did not yield significant results. Seed‐based functional connectivity analysis was performed with the seed based on differences in PCC/precentral gyrus activation between OPRM1 genotypes during evoked pain across groups. G‐allele carriers displayed decreased functional connectivity between PCC/precentral gyrus and prefrontal cortex.

**Conclusions:**

G‐allele carriers showed increased activation in PCC/precentral gyrus but decreased functional connectivity with the frontal control network during pressure stimulation, suggesting different pain modulatory processes between OPRM1 genotypes involving altered fronto‐parietal network involvement. Furthermore, our results suggest that the overall effects of the OPRM1 G‐allele may be driven by FM subjects.

**Significance:**

We show that the functional polymorphism of the μ‐opioid receptor gene OPRM1 was associated with alterations in the fronto‐parietal network as well as with increased activation of posterior cingulum during evoked pain in FM. Thus, the OPRM1 polymorphism affects cerebral processing in brain regions implicated in salience, attention, and the default mode network. This finding is discussed in the light of pain and the opioid system, providing further evidence for a functional role of OPRM1 in cerebral pain processing.

## 1 INTRODUCTION

Fibromyalgia (FM) is a chronic musculoskeletal pain disorder characterized by chronic widespread pain, accompanied by tenderness and fatigue, disturbed sleep and psychological distress. FM is considered a nociplastic pain condition (Kosek et al., 2016) accompanied by altered nociception and changes in the central nervous system (Sluka & Clauw, 2016). Importantly, FM has been associated with impaired pain inhibition (Kosek & Hansson, 1997; Lannersten & Kosek, 2010), also displayed in reduced activation of opioid‐rich regions of the pain modulatory system (Jensen et al., 2009, 2012). Additionally, elevated endogenous opioids in the cerebrospinal fluid (Baraniuk et al., 2004) could be linked to reduced μ‐opioid receptor (MOR) availability in cerebral pain‐related areas (Harris et al., 2007). The aberration in the opioid system of FM patients is in accordance with the reports of microglia activation (Albrecht et al., 2019), as opioid‐induced hyperalgesia has been associated with glial activation (Roeckel et al., 2016). Finally, the interaction between endogenous opioids and MORs has been found to influence the pain experience (Zubieta et al., 2001) and play a role in chronic pain syndromes (Zorina‐Lichtenwalter et al., 2016), including FM (Schrepf et al., 2016).

Among pain‐relevant genetic polymorphisms is the MOR gene (OPRM1), which is of interest for pharmacogenetic research investigating opioids. The functional single nucleotide polymorphism A118G *(rs1799971)* of OPRM1 leads to an exchange of asparagine to aspartic acid at amino acid 40 and affects the putative N‐terminal site of the receptor. Although inconsistent findings have been reported, carriers of at least one G‐allele, compared to AA homozygotes, have been shown to exhibit higher receptor affinity for β‐endorphins but not for other endogenous opioids or opioid drugs (Mura et al., 2013). Additionally, the G‐allele has been associated with diminished MOR expression (Bond et al., 1998; Oertel et al., 2012; Zhang et al., 2005), reduced MOR availability (Oertel et al., 2012; Peciña et al., 2015), as well as decreased MOR G‐protein coupling efficacy and, thus, reduced signalling efficacy (Oertel et al., 2009). The G‐allele has also been associated with reduced analgesic efficacy of opioid drugs (Cajanus et al., 2014; Yu et al., 2019).

Several studies indicate that the OPRM1 polymorphism affects cognition, as G‐allele carriers exhibit higher reactivity to social rejection (Way et al., 2009), lower placebo‐induced opioid activation (Oertel et al., 2009) and aberrant response to reward in healthy individuals (Lee et al., 2011) and FM subjects (Finan et al., 2010). Moreover, an antagonistic interaction between OPRM1 and serotonin‐related genes on pain modulation was observed. Specifically, exercise‐induced hypoalgesia was pronounced in FM subjects and healthy controls (HC) with the OPRM1 G‐allele combined with genetically inferred weak serotonergic mechanisms (Tour et al., 2017). Taken together, previous studies indicate that the OPRM1 polymorphism exerts an effect in acute and FM pain.

To our knowledge, no studies have investigated the role of the functional genetic polymorphism of OPRM1 in evoked pain using fMRI in FM subjects. Here, we investigated the effect of OPRM1 (*rs1799971*) on symptom severity, pain sensitivity, and cerebral processing in FM subjects and HC.

## 2 METHODS

### 2.1 Sample

The recruited study sample consisted of 80 FM subjects (mean 47.4 ± 7.9 years) and 40 HC (mean 47.9 ± 7.9 years). Data of one HC and one FM were excluded due to undetermined genotyping (see Section 2.3). Complete data sets of 79 FM patients and 39 HC were included in the behavioural analyses (*n* = 118).

Imaging data of 13 participants were excluded from further analysis due to excessive head motion (*n* = 6, see Section 2.6), structural brain anomalies (*n* = 1), and incomplete data sets due to technical issues and drop‐outs (*n* = 6), resulting in data of 105 participants included in the final fMRI analysis (70 FM subjects and 35 HC).

All patients underwent systematic screening by a specialist in rehabilitation medicine and pain relief (Dr. Kadetoff) to ensure that the ACR‐1990 as well as the ACR‐2011 classification criteria for FM (Wolfe et al., 1990, 2011) were met. Inclusion criteria for patients also included female sex, working age (20–60 years) and right‐handedness. Exclusion criteria consisted of other dominant pain conditions than FM, painful osteoarthritis, rheumatic, or autoimmune diseases, other severe somatic diseases (neurological, cardiovascular, cancer, diabetes mellitus etc.), hypertension (>160/90 mmHg), previous brain or heart surgery, psychiatric disorders including ongoing treatment for depression or anxiety, substance abuse, pregnancy, magnetic implants, self‐reported claustrophobia, obesity (Body Mass Index > 35), smoking (>5 cigarettes/day), inability to speak and understand Swedish, medication with antidepressants or anticonvulsants, inability to refrain from analgesics, NSAID or hypnotics prior to study participation (48 hr before the first visit, and 72 hr before the second visit, that is, the fMRI examination). No FM subjects were on strong opioids. HC were right‐handed women, age‐balanced to FM subjects, and in addition to the listed exclusion criteria for FM patients also free from chronic pain conditions and without regular medications with NSAIDs, analgesics or sleep medication.

Participants were recruited through advertisement in the daily newspaper. All participants received remuneration for participation and provided written informed consent before being included in the study. The study complied with the principles of the Declaration of Helsinki and was approved by the Swedish Ethical Review Authority board (permit 2014/1604‐31/1).

Note that this study is part of a larger project (see study plan https://osf.io/8zqak) including additional imaging methods and paradigms investigating pain processing in FM (Albrecht et al., 2019). One goal of the overall project was to investigate conditioned pain responses in FM subjects (Sandström et al., 2020), which have previously displayed deficits in conditioning and contingency learning (Jenewein et al., 2013; Meulders et al., 2018). Given the need to ensure successful pain conditioning in a sufficient number of participants, a larger number of FM subjects than HC was included in the project, resulting in different group sizes also in the current study.

### 2.2 Procedure

Data were collected for each participant over two consecutive days: on the first day all participants received information about the study procedure and provided saliva samples for genotyping. All participants filled out questionnaires regarding pain catastrophizing (Pain Catastrophizing Scale, PCS) (Sullivan et al., 1995), depression (Beck's Depression Inventory, BDI) (Beck et al., 1961), anxiety (State‐Trait Anxiety Inventory, STAI) (Spielberger et al., 1983) and health‐related quality of life with a focus on the bodily pain subscale (SF‐36 bodily pain) (Ware & Sherbourne, 1992). FM subjects also completed the fibromyalgia impact questionnaire (FIQ) (Burckhardt et al., 1991).

The PCS measures pain catastrophizing tendencies on a 13‐item scale, with higher scores suggest higher catastrophizing about pain. The BDI is a 21‐item test that assesses depression with higher scores indicating more depressive severity. The STAI‐State subscale consists of 20 items assessing the current state of anxiety with a score ranging from 20 to 80 with higher scores indicative of higher levels of momentary anxiety. The SF‐36 consists of eight scales with bodily pain as a two‐item subscale resulting in a final score ranging from 0 (severe, limiting pain) to 100 (no pain or limitations due to pain). The FIQ is a questionnaire assessing FM‐specific symptoms and disability. It consists of 20 items with a score ranging from 0 to 100, where a higher value indicates a poorer state of health.

Pressure pain thresholds (PPTs) were determined in all participants to assess pain sensitivity. The pressure algometer (Somedic Sales AB) was handheld and had a round 1 cm^2^ hard rubber probe that was applied perpendicular to the surface of the tested body part. The manual force was applied at a steady rate (approximately 30 kPa/s) until the participant's pain threshold was reached (Kosek et al., 1993). PPTs were collected bilaterally across four different sites: m. supraspinatus, elbow (lateral epicondyle), m. gluteus, and knee (at the medial fat pad proximal to the joint line) with one assessment per anatomical site. The average PPT across body sites is reported.

Pressure pain was applied to participants’ left calf using a cuff (13 × 85 cm) attached to a rapid cuff inflation system (E20/AG101; Hokanson). This method of deep tissue pain stimulation was chosen to provide higher ecological validity than, for example, cutaneous noxious stimulation, given that FM is characterized by widespread tissue pain. Applying pressure pain using a cuff inflator was similarly used in FM subjects before, for example, (Loggia et al., 2014).

In a comprehensive procedure, stimulus pressure intensity was individually calibrated to match ratings of 10 and 50 mm, respectively, on a visual analogue scale (VAS) ranging from 0 mm (no pain) to 100 mm (strongest imaginable pain), indicated as P10 and P50 throughout this paper. Participants were presented with a series of 5 s stimulations in increasing intensity steps of 25 mmHg in order to determine cuff PPT (first VAS rating >0) and the stimulation maximum (first VAS rating >60). In two following series five stimuli were presented in a randomized manner to determine the individual representation of P10 (starting from the PPT) and P50 (starting from the stimulation maximum). The randomized series to determine P10 used the PPT as a starting point and −2 steps and +2 steps of 25 mmHg. The randomized series to determine P50 used the stimulation maximum as a starting point and −4 steps of 25 mmHg. If the first subjective rating of 10 mm VAS was <100 mmHg, steps of 10 mmHg were used for the randomized series determining P10. Next, subjects were trained in front of a computer monitor to associate green circle with their individually calibrated P10 stimulation and a red circle with their individually calibrated P50 stimulation (familiarization phase), presented in a pseudo‐randomized order (10 × P10; 10 × P50). Following each stimulus, subjects rated their perceived pain on a 100 mm VAS.

**FIGURE 1:**
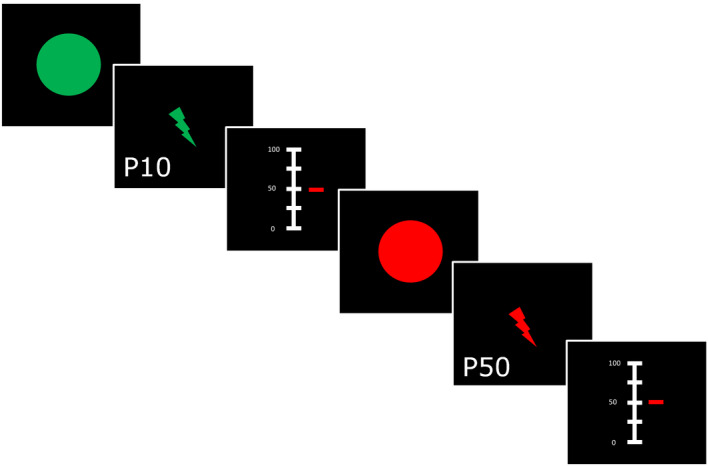
Schematic representation of the experimental paradigm. In the instructed conditioning paradigm participants were presented with a green or red cue (2 s) that was followed by a delay (2–6 s) before pressure stimulation (5 s) of lower or higher intensity, respectively. Each stimulus presentation was followed by a rating period (8 s) using a 0–100 visual analogue scale (VAS). Intensity of pressure stimulations were individually calibrated to represent approximately 10/100 VAS (P10) and 50/100 VAS (P50). Figure adapted from (Sandström et al., 2020)

On the second day, participants underwent a pressure pain paradigm during fMRI in which colour cues predicted the following stimulus intensity (Figure 1). As in the training session on the first day, a green circle was followed by the individually calibrated lower intensity pressure stimulus (10/100 VAS, P10) and a red circle was followed by the individually calibrated higher intensity stimulus (50/100 VAS, P50). Both predicting cues, green and red, and subsequent pressure stimulations, P10 and P50, respectively, were each presented 10 times in a pseudo‐randomized manner, resulting in 20 stimuli altogether. Participants were prompted to rate perceived pain intensity on a VAS after each stimulus application.

### 2.3 Genotyping

Saliva samples were collected from all participants and used for genotyping, which was performed blind to phenotypic information. In accordance with previous studies, A118G single nucleotide polymorphism *rs1799971* OPRM1 genotypes were split into two groups, AA versus AG/GG (Mura et al., 2013; Tour et al., 2017). Genotyping was performed using TaqMan single nucleotide polymorphism genotyping assays and ABI 7,900 HT instrument (Applied Biosystems [ABI]). Polymerase chain reactions (PCRs), with a total volume of 5 ml, were performed in 384‐well plates containing 2.5 ml Universal Master Mix and 5 ng dried‐down genomic DNA per well. The PCR amplification protocol included two holds, 50°C for 2 min and denaturation at 95°C for 10 min, followed by 50 cycles at 92°C for 15 s and 60°C for 1 min. In one FM and one HC the genotype could not be determined, as the PCRs did not produce secure read‐outs.

### 2.4 Statistical analysis of behavioural data

Behavioural data analysis was performed with R (RStudio Team, 2016) and included 118 participants (FM patients *n* = 79, HC *n* = 39) (Table 1). A chi‐squared test and fisher's exact test were used to analyse genotype frequencies and to assess deviations from Hardy–Weinberg equilibrium.

**TABLE 1:** Characteristics of OPRM1 genotypes reported for fibromyalgia (FM) subjects and healthy controls (HC) (*n* = 118)

	OPRM1 AA (*n* = 88)		OPRM1 */G (*n* = 30)	
	FM (*n* = 57; ~72% of FM)	HC (*n* = 31; ~79% of HC)	FM (*n* = 22; ~28% of FM)	HC (*n* = 8; ~21% of HC)
Mean age in years (*SD*)	46.4 (±8)	47.3 (±7.8)	48.7 (±7.6)	50 (±8.7)
Median input pressure in mmHg (mean, *SD*)	P10: 60 (71, ±38)	P10: 125 (128, ±38)	P10: 68 (69, ±28)	P10: 100 (104, ±38)
	P50: 175 (194, ±71)	P50: 295 (295, ±64)	P50: 192 (201, ±88)	P50: 255 (263, ±74)
Mean PPTs in kPa (*SD*)	153 (±58.1)	323 (±118)	154 (±73.3)	293 (±86.6)
Mean PCS scores (*SD*)	17.8 (±10.9)	4.1 (±5.9)	18.8 (±11.2)	6.9 (±11.6)
Mean BDI scores (*SD*)	16 (±8)	0.2 (±0.67)	15.1 (±7.8)	0.3 (±0.46)
Mean STAI‐S scores (*SD*)	43.3 (±11.8)	29.4 (±7.1)	44.5 (±12.3)	32.2 (±9.7)
Mean SF−36 bodily pain scores (*SD*)	30.2 (±14.8)	89.5 (±11.8)	33.6 (±14.3)	85.9 (±16.2)
Mean FIQ scores (*SD*)	63.6 (±16.2)	–	61.5 (±18.3)	–
Mean pain duration in months (*SD*, min, max)	184 (±112, 24, 492)	–	189 (±89.2, 60, 408)	–

Abbreviations: BDI, Beck's Depression Inventory; FIQ, fibromyalgia impact questionnaire; kPa, kilopascal; max, maximum; min, minimum; mmHg, millimetres of mercury; PCS, pain catastrophizing scale; *SD*, standard deviation; STAI‐S, State‐trait anxiety inventory (state).

To assess differences in calibrated input pressure, a linear mixed effects model (using *nlme*, Pinheiro et al., 2020) with fixed effects pressure level (P10/P50), group (FM/HC) and OPRM1 genotype (AA/*G) including all interactions was used. Variability between participants was accounted for including random intercepts and by‐subject‐over‐pressure level random slopes accounted for individual variability between pressure level. Restricted maximum likelihood was used to estimate variances of random effects, different variances were allowed for each level of the factors OPRM1 genotype and group, and a first‐order autoregressive correlation structure was modelled to account for intra‐subject dependencies in repeated measures.

Subjective pain ratings acquired throughout the fMRI paradigm were analysed using another linear mixed effects model. Mixed model analysis was performed with the fixed effects OPRM1 variants, group, pressure level, PPTs and the continuous variable time with random intercepts, accounting for variability between participants, and by‐subject‐over‐time random slopes, accounting for individual variability over time. Interaction effects on pain ratings between OPRM1 genotype and time, group and pressure levels were also tested. As in the mixed model on pressure intensity, restricted maximum likelihood was used to estimate variances of random effects, different variances were allowed for each level of the factors OPRM1 genotype and group, and a first‐order autoregressive correlation structure was modelled to account for intra‐subject dependencies in repeated measures.

In order to investigate the effect of clinical and pain‐relevant variables on pain ratings, another mixed model analysis was performed using only FM subject data. Here, PCS, BDI, FIQ, and SF‐36 bodily pain scores were included to test for a potential association with experimental pain ratings acquired throughout the paradigm. Apart from the additionally included FM‐relevant variables, this model was set up as previously described (without the factor group).

A two‐way ANOVA with the factors genotype and group was used to test for differences in age. Given the variance differences in groups in clinical measures, that is, PCS, BDI, STAI‐State, SF‐36 bodily pain scores and PPTs, robust two‐way ANOVAs with trimmed means (trim level = 0.2) were performed (using *WRS2*, Mair & Wilcox, 2020). STAI data of one FM subject and PPTs of one FM subject were missing at random. Welch tests were performed to test for differences in FM pain duration and FIQ scores between OPRM1 genotypes in FM subjects. A *p* < 0.05 was considered statistically significant in all analyses.

### 2.5 MRI data acquisition

fMRI data were collected on a 3T Scanner (General Electric 750) using an eight‐channel head coil. Functional images comprised 42 axial slices (slice thickness 3 mm, 0.5 mm gap) and were acquired using a T2*‐sensitive gradient echo‐planar imaging sequence (TR 2 s; TE 30 ms; flip angle 70°; field of view 220 × 220 mm, 72 × 72 mm matrix; 3 × 3 mm in‐plane resolution). The first five volumes were discarded to account for stabilization of the T1‐relaxation effects. Prior to the functional sequence, high‐resolution T1‐weighted anatomical images were acquired (BRAVO, voxel size 1 × 1 × 1 mm, 176 slices).

### 2.6 Analysis of fMRI data

Processing and analysis of functional data was performed using statistical parametric mapping (SPM12; Wellcome Trust Centre for Neuroimaging) running under MATLAB (The MathWorks, version R2015b). Data of 105 participants (FM = 70, HC = 35) were included in the fMRI analysis (Table S1).

First, anatomical and functional scans were reoriented manually to the anterior commissure. Functional images were spatially realigned to the mean volume using a six‐parameter affine transformation. Then, the anatomical T1‐weighted image was co‐registered to the functional images. Functional images were spatially normalized into Montreal Neurological Institute (MNI) stereotactic standard space and smoothed with a 6 mm full‐width at half‐maximum isotropic Gaussian kernel. Framewise displacement (FD) was used to assess head movement from one frame relative to the previous by calculating the sum of the absolute values of the derivatives of the six realignment parameters (Power et al., 2012). As a consequence, six participants (four FM subjects, two HC) were excluded from further analyses due to excessive head motion (FD > 0.5 in >15% of the images). There were no differences in FD between FM and HC (Wilcoxon rank sum test, *Z* = 1.58, *p* = 0.1145). The general linear model as implemented in SPM12‐7219 was used for subsequent data analysis. First level analysis included temporal high‐pass filtering (cut‐off 128 s) and correction for auto‐correlations using first‐order autoregressive modelling. The following conditions were modelled on the individual level: pressure stimulations for two intensities (P10/P50, 5 s), two cue‐anticipation phases (red preceding P50/green preceding P10, 2 s cue plus delay of 2–6 s before stimulus onset) and rating period (8 s). Six realignment‐derived motion parameters were added as regressors of no interest. In order to link variations of pain intensity perception to neural activity, additional first level models were specified that included individual pain ratings for each stimulus as a parametric modulator of the regressors representing P50 and P10. Single‐subject contrast images were then taken to second level random‐effects analyses with unequal variances between groups and genotypes being assumed.

Our functional imaging analysis aimed to test whether the genetic polymorphism of OPRM1 affects pain‐related processing in FM subjects and HC and examine which brain regions may be functionally connected to areas differing between genotypes. In addition, we explored whether OPRM1 influences processing during the cue‐anticipation phase (red preceding P50/green preceding P10), as an indicator for anticipatory and/or psychological processes. First, the effect of painful pressure stimulation and cue‐anticipation was tested separately for each group and pressure level (P10/P50). To test for a possible interaction between OPRM1 genotype and pressure level as well as group and pressure level, two‐sample *t* tests were performed using individual contrast images of (a) pressure intensity (P50‐P10) and (b) cue‐anticipation colour (red preceding P50‐green preceding P10). Groups and genotypes were contrasted separately for each cue‐anticipation colour and during noxious stimulation using parametric trial‐by‐trial responses. An OPRM1 genotype‐by‐group interaction during noxious stimulation as well as during cue‐anticipation was tested using full factorial models.

A region‐of‐interest (ROI) approach was used to test whether differences between OPRM1 genotypes are present in opioid‐rich brain areas, that is, regions demonstrating a correlation between MOR availability/binding potential and blood oxygen level dependent (BOLD) signal in FM subjects during evoked pain (Schrepf et al., 2016). ROIs were based on findings by Schrepf and colleagues using anatomical masks derived from the Harvard‐Oxford Atlas freely distributed with FSL (https://fsl.fmrib.ox.ac.uk/fsl/fslwiki/Atlases). The probability maps, namely left posterior cingulate cortex (PCC), right precentral gyrus (encompasses the primary motor cortex, M1), left anterior cingulate cortex (ACC), and left middle temporal gyrus (temporo‐occipital part) were conservatively thresholded at 50%. Note that the right ACC mask was thresholded at 25%, as a more conservative threshold excluded the subgenual portions, which were reported by Schrepf et al. (2016). Also note that the dorsolateral prefrontal gyrus (DLPFC) is not an anatomical region per se but the coordinates reported by Schrepf were best represented by the left middle frontal gyrus (thresholded at 25%). The mask for the cerebellum was also based on the provided coordinates located in crus II based on the SUIT cerebellum atlas (http://www.diedrichsenlab.org/imaging/propatlas.htm). Given that the ROIs were based on data from FM subjects, differences between OPRM1 genotypes were first tested in FM subjects. For completion, and after testing for an interaction effect with group, the analysis was also performed on pooled data. To test for a main effect of OPRM1 within ROIs, two‐sample *t*‐tests were performed between OPRM1 genotypes for painful pressure stimulations (P10 + P50, that is, pain > baseline). Next, OPRM1 variants were contrasted in a two‐sample *t* test using a whole brain approach to identify regions displaying functional differences outside of opioid‐rich brain areas included in the ROI approach. To further investigate contributions to OPRM1 genotype differences, we explored differences between OPRM1 variants separately in each group.

A psychophysiological interaction analysis (PPI) analysis (Friston et al., 1997) was performed to identify differences in functional connectivity associated with the OPRM1 polymorphism pooled across groups. A PPI analysis tests for an interaction between a predetermined seed region in the brain (physiological factor) with other brain areas during an experimental condition (psychological factor). Here, individual time series were extracted from the cluster showing differences in activation between OPRM1 genotypes during evoked pain across group and pressure levels (peak at [−2 –28 48]). We then contrasted the OPRM1 genotypes to identify differences in coupling with other brain regions related to the differential processing of evoked pain between genetic variants.

Extracted betas from the cluster found to differ between OPRM1 variants in FM subjects were correlated with clinical and pain‐relevant factors for each OPRM1 genotype. Previously, PCC activity has been shown to be increased during FM pain catastrophizing (Lee et al., 2018) and has been associated with trait pain catastrophizing (Galambos et al., 2019). For this reason, betas extracted from the ROI PCC analysis were correlated with PCS scores in FM subjects in an additional analysis.

For all fMRI analysed the initial statistical threshold was set to *p* < 0.001 and a cluster threshold of *p* < 0.05 (family wise error corrected) was applied, unless otherwise reported. Results are presented stating *x*, *y*, *z* coordinates in MNI space.

## 3 Results

### 3.1 Behavioural data results

#### 3.1.1 Demographics and questionnaires

The participant characteristics are described in Table 1. As expected, FM subjects scored significantly higher than HC on PCS (*Q* = 67.743, *p* < 0.001), BDI (*Q* = 180.33, *p* < 0.001), STAI (*Q* = 28.862, *p* < 0.001) and SF‐36 bodily pain (*Q* = 412.284, *p* < 0.001). There was neither a difference between OPRM1 variants nor an interaction between genotype and group with respect to BDI, PCS, STAI, SF‐36 bodily pain and age (all *p* > 0.3). In FM subjects, there was no difference between OPRM1 genotypes in pain duration (*t*(44.7) = −0.193, *p* = 0.848) and FIQ scores (*t*(34.5) = 0.479, *p* = 0.635).

#### 3.1.2 Genotype frequencies

The OPRM1 genotype frequency, that is, the distribution of homozygous AA and G‐allele carriers, in the sample did not deviate from the Hardy–Weinberg equilibrium, (*χ* ^2^(1) = 2.17, *p* = 0.141) and was similar in FM subjects and HC (*χ* ^2^(1) = 0.546, *p* = 0.46).

#### 3.1.3 Pressure pain thresholds

As expected, FM subjects had significantly lower PPTs than HC (*Q* = 50.86, *p* < 0.001), indicating higher pain sensitivity. No difference between OPRM1 genotypes or interaction between group and genotype was observed in PPTs.

#### 3.1.4 Input pressure

**FIGURE 2:**
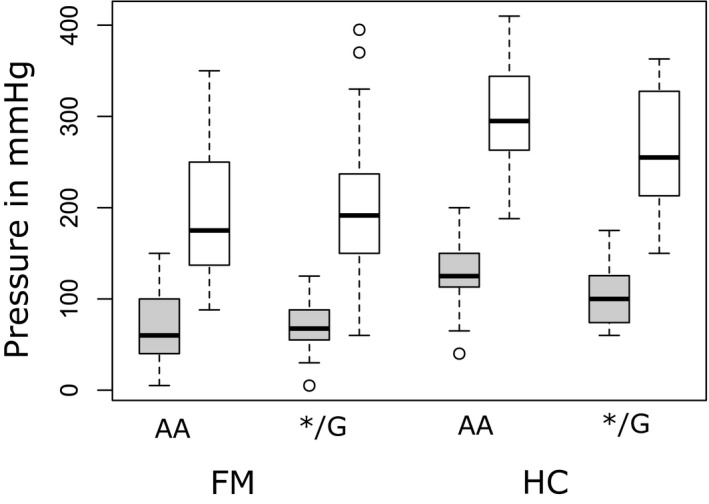
Calibrated pressure input in mmHg for OPRM1 genotypes (*/G vs. AA) to match 10/100 VAS (P10) and 50/100 VAS (P50) pressure intensity. There was a significant difference in input pressure (i) between groups and (ii) between P50 (white boxplot) and P10 (grey boxplot) pressure in mmHg. No difference between OPRM1 genotypes was observed. Whiskers represent the maximum 1.5 interquartile range (IQR). Circles represent data outside the IQR. FM, fibromyalgia; HC, healthy controls; VAS, visual analogue scale

In line with previous findings, FM displayed increased pain sensitivity compared to HC by requiring lower input pressure stimuli for pain intensities equivalent to P10 and P50 (*β* = 56.717 [95% confidence interval (CI) 39.9, 73.4], *t* = 17.38, *SE* = 8.445, *p* < 0.001). In addition, a main effect for the input pressure was found (*β* = 123.731 [CI 109.6, 137.8], *t* = 6.716, *SE* = 7.119, *p* < 0.001). The observed main effects group and input pressure were qualified by statistically significant group × pressure intensity interaction (*β* = 40.331 [CI 17.3, 63.4], *t* = 3.469, *SE* = 11.625, *p* < 0.001). There were no differences between OPRM1 genotypes or interactions including the OPRM1 variant (all *p* > 0.2) (Table 1; Figure 2).

#### 3.1.5 Pain ratings

**FIGURE 3:**
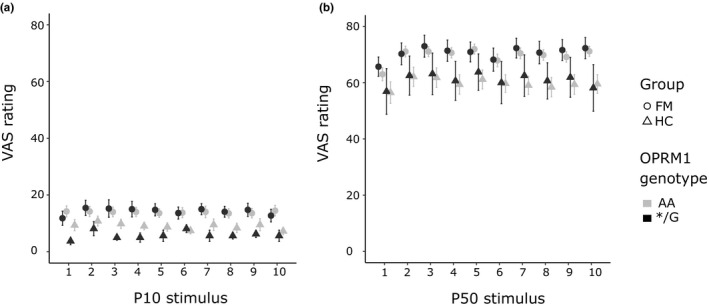
Pain ratings for OPRM1 genotype A (*n* = 88) and OPRM1 G‐carriers (*n* = 30) during 10/100 VAS (P10) and 50/100 VAS (P50) stimulus pressure intensity. Individual pain ratings were acquired using a VAS ranging from ‘no pain’ (0) to ‘worst pain imaginable’ (100). There was a significant difference between groups (FM/HC) and between stimulus intensities (P10/P50) but no difference between OPRM1 genotypes (AA/*G). (a) Pain ratings are displayed for stimuli of lower intensity (P10) and (b) for stimuli of higher intensity (P50). Error bars represent the standard error of the mean. FM, fibromyalgia; HC, healthy controls; VAS, visual analogue scale

There was a difference in pain ratings between stimulus intensities (P10/P50), indicating successful calibration within participants (results are presented in Table 2; Table S2). Pain ratings also differed between groups, indicating FM subjects rated pain intensity higher than HC, despite individual pressure intensity calibration (Figure 3). No difference in ratings over time or between OPRM1 genotypes was observed. No interactions between OPRM1 genotype × stimulus intensity, time or group were found in pain ratings (Figure 3).

**TABLE 2:** Linear mixed model results for predictors of pain ratings

Effect	*β*	Lower CI	Upper CI	*SE*	*t‐*value	*p*‐value
Group	−7.407	−13.217	−1.596	2.933	−2.526	0.013*
OPRM1 genotype	1.643	−5.52	8.807	3.615	0.454	0.65
Pressure level	−51.052	−53.483	−48.622	1.239	−41.196	<0.001*
Time	0.135	−0.082	0.352	0.111	1.217	0.224
PPT	−0.006	−0.023	0.016	0.011	−0.509	0.612
OPRM1 × group	−1.411	−9.21	6.388	3.936	−0.359	0.721
OPRM1 × pressure level	−3.565	−7.562	0.432	2.038	−1.749	0.081
OPRM1 × time	−0.002	−0.404	0.399	0.205	−0.009	0.993

Abbreviations: CI, 95% confidence interval; PPT, pressure pain threshold; SE, standard error;*β*, beta estimate.

* *p* < 0.05

Given differing group sizes and genotype distributions, Cook's distance was calculated across groups to identify potentially influential data points (118 subjects × 20 ratings, that is, 2,360 observations) in the model. Cook's distance was overall small (mean = 2.03e‐04, median = 6.213e‐05), indicating single data points did not affect the model in a substantial way. Nonetheless, a commonly used cut‐off value of 4/number of observations was adopted to determine potentially influential data points, resulting in 42/2,360 observations (1.78%) surpassing the threshold (1.69e‐03). In order to investigate the influence of these observations, the mixed model was repeated without influential data points, yielding similar results.

In FM subjects, there was a significant difference in pain ratings between stimulus intensity (P10/P50) but no effect of OPRM1 genotype, time or interaction between them (results are presented in Table 3). Of the included pain‐related measures, only PCS showed a significant effect, with higher PCS scores being linked to higher experimental pain ratings. There was no evidence of PPTs, BDI, FIQ, or SF‐36 bodily pain scores influencing experimental pressure pain ratings in FM subjects.

**TABLE 3:** Linear mixed model results for predictors of pain ratings in fibromyalgia subjects

Effect	*β*	Lower CI	Upper CI	*SE*	*t‐*value	*p*‐value
OPRM1 genotype	2.136	−5.154	9.425	3.655	0.584	0.561
Pressure level	−52.439	−55.483	−49.445	1.526	−34.355	<0.001*
Time	0.23	−0.04	0.499	0.137	1.673	0.095
PPT	−0.014	−0.049	0.021	0.018	−0.789	0.433
PCS	0.321	0.065	0.559	0.124	2.523	0.014*
BDI	−0.253	−0.607	0.101	0.177	−1.428	0.158
FIQ	0.009	−0.172	0.19	0.091	0.099	0.921
SF‐36 bodily pain	−0.057	−0.251	0.138	0.098	−0.581	0.563
OPRM1 × pressure level	−3.199	−7.908	1.511	2.401	−1.332	0.183
OPRM1 × time	−0.071	−0.547	0.406	0.243	−0.291	0.771

Abbreviations: BDI, Beck's Depression Inventory; CI, 95% confidence interval; FIQ, fibromyalgia impact questionnaire; PCS, pain catastrophizing scale; PPT, pressure pain threshold; SE, standard error;*β*, beta estimate.

* *p* < 0.05

As in the previous model, Cook's distance in the FM model was overall small (mean = 2.49e‐04, median = 8.01e‐05), with 17 out of 1,580 observations (1.08%) surpassing Cook's distance of 4/number of observations (2.53e‐03). A comparison of mixed models, with and without potentially influential data points, yielded comparable results.

Analysing only data of participants included in the fMRI analysis (*n* = 105) revealed similar results in all behavioural analyses (see Tables S1 and S2; Figure S1).

### 3.2 Functional imaging results

#### 3.2.1 BOLD responses to painful pressure stimulation

**FIGURE 4:**
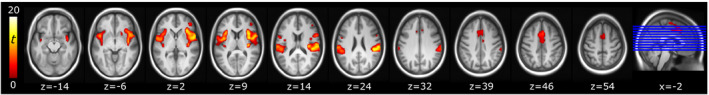
Main effect of pressure pain stimulation. A main effect of pressure stimulus was observed in regions associated with pain processing, including insula and somatosensory cortices/parietal operculum. Maps are displayed whole brain family wise error‐corrected at a threshold of *p* < 0.05 using a one‐sample *t* test resulting in *t*‐maps, overlaid on a group‐average structural image

Analysis of fMRI data showed increased BOLD response during painful pressure stimulation in brain regions commonly associated with pain processing (Apkarian et al., 2005; Peyron et al., 2000), including insula, postcentral gyrus (primary somatosensory cortex, S1) and parietal operculum (secondary somatosensory cortex, S2) (Figure 4; Table 4). Neural activity linked to pain ratings using parametric modulation displayed increased activation in operculum, ACC, postcentral gyrus, and thalamus (Table 4). BOLD responses during pressure stimulation separately in both FM subjects and HC for each pressure level (P10/P50) are presented in Table S3.

**TABLE 4:** Localization of significant clusters (*p* < 0.05) during noxious stimulation (pain > baseline) across participants (Figure 4) and using experimental pain ratings as a parametric modulator

Region/s	Peak coordinate			*t*	Cluster size (*k*)	*p*‐value
	*x*	*y*	*z*			
Pressure stimuli > baseline^a^						
R parietal operculum	46	−28	20	21.24	4,880	<0.001
R parietal operculum/R supramarginal gyrus	58	−24	22	18.24		
R central operculum	54	4	4	17.53		
R postcentral gyrus	16	−38	70	15.84	2,580	<0.001
R postcentral gyrus	8	−38	64	12.77		
R precentral gyrus	6	−22	64	12.52		
L parietal operculum	−52	−34	20	14.69	3,249	<0.001
L insula/L central operculum	−38	0	10	14.42		
L frontal operculum/L insula	−34	16	8	13.64		
R putamen	18	14	−6	8.62	59	<0.001
R frontal pole	44	42	2	8.14	171	<0.001
R middle frontal gyrus	44	38	18	6.03		
Pressure stimuli > baseline with parametric modulator pain ratings						
R central operculum	60	0	6	6.42	3,034	<0.001
R temporal pole/R superior temporal gyrus	60	8	−2	5.92		
L planum polare	−60	−4	4	6.01	2,504	<0.001
L planum polare/L central operculum	−56	2	−2	5.65		
L postcentral gyrus	−44	−14	30	5.33		
R ACC	4	−2	46	5.98	1,234	<0.001
L juxtapositional lobule cortex	−2	−10	64	4.86		
R juxtapositional lobule cortex	2	−4	58	4.78		
L thalamus	−14	−22	4	4.02	227	0.009
L thalamus	−12	−14	0	3.68		
R thalamus	20	−22	6	3.63		

Note

Anatomical site, maximum *t* value and MNI coordinates (in mm) of the local maxima. Parietal operculum = S2; postcentral gyrus = S1; precentral gyrus = M1.

Abbreviations: ACC, anterior cingulate cortex; L, left; R, right.

^a^ Reported at *p* < 0.05, whole brain family wise error‐corrected.

#### 3.2.2 Group differences

Parametric response did not differ between groups, providing no evidence of differing pain processing in individually calibrated pressure intensities.

#### 3.2.3 ROI analyses of OPRM1 differences during painful pressure stimulation

First, we focused on ROIs that had previously shown a correlation between MOR availability/binding potential and BOLD signal during evoked pain in FM subjects, that is, left middle frontal gyrus/DLPFC, left perigenual ACC, left middle temporal gyrus, left PCC, right subgenual ACC and cerebellum (Schrepf et al., 2016). Here, there were significant differences between OPRM1 genotypes in PCC and precentral gyrus (both thresholded at 50%) but not in other predetermined ROIs in FM subjects. Specifically, FM OPRM1 G‐carriers showed increased activation compared to AA homozygotes in PCC and precentral gyrus (Table 5), two opioid‐rich brain regions previously shown to be functionally connected to pain‐evoked BOLD signal in FM subjects.

**TABLE 5:** Localization of significant clusters (*p* < 0.05) in region‐of‐interest analysis (based on Schrepf et al., 2016) during noxious stimulation (pain > baseline)

Region/s	Peak coordinate			*t*	Cluster size (*k*)	*p*‐value
	*x*	*y*	*z*			
FM OPRM1 */G > OPRM1 AA						
L PCC	−2	−28	46	4.59	16	0.033
FM OPRM1 */G < OPRM1 AA						
N/S						
FM OPRM1 */G > OPRM1 AA						
R Precentral gyrus	2	−26	66	3.93	22	0.049
FM OPRM1 */G < OPRM1 AA						
N/S						
OPRM1 */G > OPRM1 AA						
L PCC	−2	−28	46	4.63	28	0.023
PCC	0	−24	36	3.41	10	0.039
OPRM1 */G < OPRM1 AA						
N/S						
OPRM1 */G > OPRM1 AA						
R Precentral gyrus	2	−28	64	4.27	48	0.023
OPRM1 */G < OPRM1 AA						
N/S						

Note

Anatomical site, maximum *t* value and MNI coordinates (in mm) of the local maxima. Precentral Gyrus = M1.

Abbreviations: FM, fibromyalgia; L, left; PCC, posterior cingulate cortex; R, right.

No group‐by‐genotype effect was observed in predetermined ROIs, indicating no systematic difference between groups with respect to OPRM1 genotype. A subsequent ROI analysis on pooled group data revealed very similar results as observed in FM subjects. Specifically, OPRM1 */G displayed increased activation compared to AA homozygotes in PCC and precentral gyrus (Table 5). No effect in the opposite direction, that is, OPRM1 AA> */G, emerged in any ROI analysis.

#### 3.2.4 Whole brain analyses of OPRM1 differences during painful pressure stimulation

For completion, we performed whole brain analyses to test for differences outside of predetermined ROIs, finding no significant interaction between genotype × pressure level (P10/P50), suggesting no substantial difference in BOLD response between the two painful pressure intensities depending on the polymorphism variant. In addition, there was no interaction between OPRM1 genotype (AA/ */G) × group (FM/HC), suggesting genotypes did not differ systematically in neural response between groups. This is in line with the findings in the previous ROI analysis, where no group‐by‐genotype interaction was observed.

**FIGURE 5:**
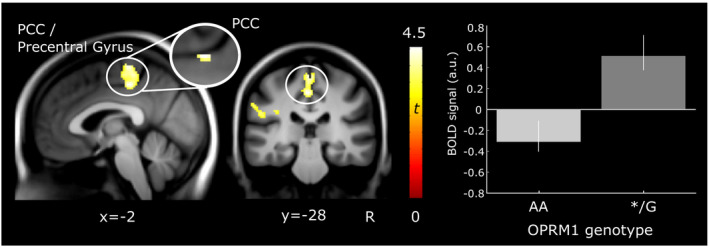
Cortical brain activity during processing of painful pressure stimulation for OPRM1 genotype A (*n* = 77) compared to OPRM1 G‐carriers (*n* = 28). Carriers of at least one G allele displayed increased activation in a cluster encompassing the posterior cingulate cortex (PCC) and precentral gyrus (peak at [−2 –28 48], Table 6) with an enlarged image of the finding from the region‐of‐interest PCC analysis (peak at [−2 –28 46], Table 5). Results are overlaid on a group‐average structural image (visualization threshold *p* < 0.001 uncorrected). The bar plot shows group means and standard errors of parameter estimates extracted from the activation cluster of the contrast OPRM1 */G > OPRM1 AA. a.u., arbitrary units; R, right

Given that no interactions between OPRM1 genotype × group as well as OPRM1 genotype × pressure level were found, we further investigated whether OPRM1 variants exert an effect on the cerebral processing of noxious stimulation (pain > baseline). We found that OPRM1 genotypes differed significantly in BOLD signal during the processing of painful stimuli in just one prominent cluster, which largely overlapped with findings from the ROI analysis. OPRM1 G‐carriers showed increased activation compared to AA homozygotes in a cluster encompassing PCC and precentral gyrus/postcentral gyrus (Figure 5). Note that while we preserved laterality in the ROI analysis (Table 5), the whole brain analysis revealed that the OPRM1 effect stretched across both hemispheres (Table 6). There was no effect in the opposite direction, that is, no increased activation in OPRM1 genotype AA compared to */G.

**TABLE 6:** Localization of significant clusters (*p* < 0.05) showing differences between OPRM1 genotypes during noxious stimulation (pain > baseline)

Region/s	Peak coordinate			*t*	Cluster size (*k*)	*p*‐value
	*x*	*y*	*z*			
Pooled OPRM1 */G > OPRM1 AA						
L PCC/L precentral gyrus	−2	−28	48	4.63	350	0.005
L precentral gyrus	−2	−26	66	4.41		
L precentral gyrus/L postcentral gyrus	−2	−32	58	4.19		
FM OPRM1 */G > OPRM1 AA						
L PCC/L precentral gyrus	−2	−28	48	4.68	223	0.034
R precentral gyrus	2	−26	66	3.67		
L precentral gyrus/L postcentral gyrus	−2	−32	58	3.69		
HC OPRM1 */G > OPRM1 AA						
N/S						

Note

Anatomical site, maximum *t* value and MNI coordinates (in mm) of the local maxima. Postcentral gyrus = S1, precentral gyrus = M1.

Abbreviations: FM, fibromyalgia; HC, healthy controls; L, left; PCC, posterior cingulate cortex; R, right.

**FIGURE 6:**
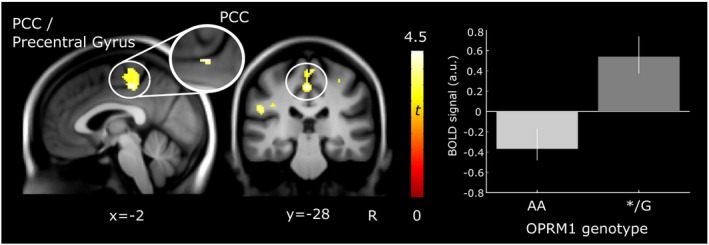
Cortical brain activity in FM subjects (*n* = 71) during processing of evoked pain for OPRM1 genotype A (*n* = 50) compared to OPRM1 G‐carriers (*n* = 21). FM carriers of at least one G allele displayed increased activation in a cluster encompassing the posterior cingulate cortex (PCC) and precentral gyrus (peak at [−2 –28 48], Table 6) with an enlarged image of the finding from the region‐of‐interest PCC analysis (peak at [−2 –28 46], Table 5). Results are overlaid on a group‐average structural image (visualization threshold *p* < 0.001 uncorrected). The bar plot shows group means and standard errors of parameter estimates extracted from the activation cluster of the contrast OPRM1 */G > OPRM1 AA. a.u., arbitrary units; R, right

To further identify contributions to the observed OPRM1 genotype differences across groups, we explored differences between OPRM1 variants in FM subjects and HC separately across the whole brain. In FM subjects (*n* = 71), we observed that G‐allele carriers displayed increased activation compared to the AA homozygotes in PCC/precentral gyrus (Figure 6; Table 6). As in the ROI approach, the observed differences between OPRM1 genotypes in FM subjects were found only in a very similar location as observed in the previous analysis including all participants. Comparing OPRM1 genotypes in HC only (*n* = 34), we did not observe differences during evoked pain at the applied threshold. No effect was found in either group for the opposite direction (OPRM1 AA > */G).

#### 3.2.5 PPI analysis

**FIGURE 7:**
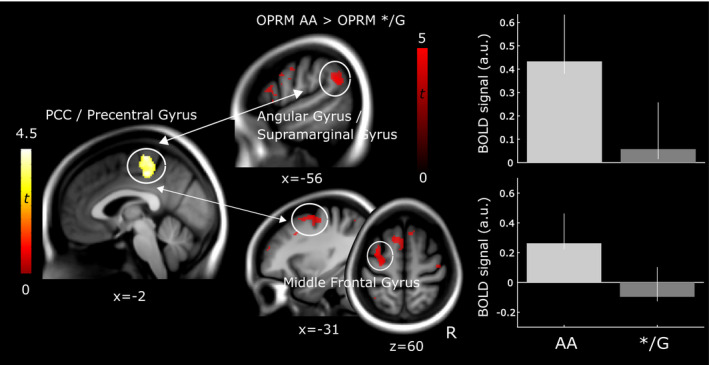
Psychophysiological interaction analysis. Psychophysiological interaction analysis revealed increased functional coupling in homozygote OPRM1 AA compared to OPRM1 G‐carriers between posterior cingulate cortex/precentral gyrus and middle frontal gyrus/DLPFC (peak at [−34 2 60], Table 7) extending to the precentral gyrus. In addition, increased coupling was observed with the inferior parietal lobe (peak at [−58 –54 30]), that is, angular gyrus extending to the supramarginal gyrus. Results are overlaid on a group‐average structural image (visualization threshold *p* < 0.001 uncorrected). The bar plots display group means and standard errors of parameter estimates extracted from the respective activation cluster. a.u., arbitrary units; R, right

On the basis of the increased activation in PCC/precentral gyrus in G‐allele carriers observed in the whole brain analysis pooled across groups (peak at [−2 –28 48]), we explored whether different functional connectivity accompanies differences in BOLD activation between genotypes. Here, we observed decreased coupling in OPRM1 genotype */G compared to AA between the described seed cluster comprised of PCC/precentral gyrus with left middle frontal gyrus/DLPFC extending to left precentral gyrus (Figure 7; Table 7). The observed target cluster encompassing the left precentral gyrus was located more lateral than the seed cluster of PCC/precentral gyrus. In addition, decreased functional connectivity in OPRM1 */G was observed from the same seed region with the left angular gyrus extending to the left supramarginal gyrus, that is, areas comprising the inferior parietal lobe (Figure 7; Table 7). There was no effect in the opposite direction, that is, no increased connectivity between PCC/precentral gyrus and other brain regions in OPRM1 */G compared to homozygous AA.

**TABLE 7:** Localization of significant clusters (*p* < 0.05) showing differences in functional connectivity during pressure pain stimulation (pain > baseline)

Region/s	Peak coordinate			*t*	Cluster size (*k*)	*p*‐value
	*x*	*y*	*z*			
OPRM1 AA > OPRM1 */G						
L middle frontal gyrus/L DLPFC	−34	2	60	5.13	1,518	<0.001
L middle frontal gyrus/L DLPFC	−46	22	38	5.08		
L precentral gyrus	−28	−8	58	5.06		
L angular gyrus/L supramarginal gyrus	−58	−54	30	4.91	183	0.033
L angular gyrus	−48	−50	40	3.74		
L angular gyrus	−54	−58	40	3.73		
OPRM1 AA < OPRM1 */G						
N/S						

Note

Anatomical site, maximum t value, and MNI coordinates (in mm) of the local maxima. Precentral gyrus = M1.

Abbreviations: DLPFC, dorsolateral prefrontal gyrus; L, left; R, right.

#### 3.2.6 OPRM1 differences during the cue‐anticipation phase

There were neither differences observed between groups in the cue‐anticipation phase nor an interaction between groups and cue‐anticipation colour (red preceding P50/green preceding P10). BOLD responses during cue‐anticipation phase for each group and cue‐anticipation colour are presented in Table S4. In addition, there was no significant difference between OPRM1 genotypes during the cue‐anticipation phase or between cue‐anticipation colour depending on OPRM1 variant. These results provide no evidence that anticipatory and/or psychological effects processes are affected by OPRM1.

#### 3.2.7 Correlation between BOLD response and clinical measures in FM subjects

Finally, extracted BOLD response in FM subjects from the observed OPRM1 effect (pain > baseline) in the cluster encompassing PCC/precentral gyrus were correlated with clinical and pain‐relevant measures for each OPRM1 genotype. No correlation for either OPRM1 genotype was observed for PPTs, SF‐36 bodily pain or FIQ (all *p* > 0.3), finding no association between BOLD response to evoked pressure pain or any clinical measures in FM subjects depending on OPRM1 genotype.

In addition, there was no correlation observed between PCC activation obtained from the ROI analysis and PCS scores. Note that using data from the same project we recently found that higher catastrophizing in FM is associated with increased BOLD response in prefrontal cortices and reduced functional connectivity between inferior parietal lobe and thalamus during pressure pain stimulation in a previously conditioned low‐pain condition (Sandström et al., 2020). Importantly, the previously reported correlation between the BOLD response and catastrophizing was located in other brain regions than the OPRM1 effect observed in the current data.

## 4 DISCUSSION

In this study, we investigated the influence of the functional polymorphism of the MOR gene (OPRM1, A118G *rs1799971*) on processing of evoked pain in FM subjects and HC using fMRI. Our data showed no systematic difference in neural response to nociceptive pressure stimulation between groups with respect to OPRM1 genotypes. Pooled across groups, we found that OPRM1 G‐carriers (AG or GG) displayed increased activation in PCC extending to the precentral gyrus, compared to AA homozygotes. This finding was observed in FM subjects alone but not in HC, indicating that FM subjects may drive the effect, even though the group‐by‐genotype interaction yielded no significant result. Across groups, decreased functional connectivity was found in OPRM1 */G compared to AA between PCC/precentral gyrus and (a) the fronto‐parietal network, that is, DLPFC/middle frontal gyrus, and (b) inferior parietal lobe, that is, angular and supramarginal gyrus. Our findings suggest that differences in pain‐evoked neural response and functional coupling may mirror differing modulatory mechanisms between OPRM1 variants.

In accordance with previous studies (Peciña et al., 2015; Tour et al., 2017), OPRM1 genotypes did not differ in pain sensitivity, suggesting that both variants similarly modulate pain but through varying routes. Schrepf and colleagues link the less reactive endogenous opioid system in FM, displayed as reduced MOR availability using positron emission tomography (PET) (Harris et al., 2007) and decreased BOLD response during evoked pain in antinociceptive brain regions (Schrepf et al., 2016). Similar to FM subjects (Baraniuk et al., 2004), OPRM1 G‐carriers could hypothetically have a higher baseline opioid tone and, thus, possess a hyporeactive opioid system when challenged by noxious stimulation, consistent with reduced placebo responses (Peciña et al., 2015). The observed differences between genotypes were located in the PCC, a key node in the default mode network (DMN) (Fransson & Marrelec, 2008), suggested to be relevant for internally directed cognition and attention (Leech & Sharp, 2014). While differences in PCC were regarded as particularly interesting, the precentral gyrus may contribute to the observed effect, for example, through the discussed role of M1 in experimental muscle pain (Burns et al., 2016). Given our results in two out of several ROIs, our findings suggest that functional consequences of OPRM1 may not be specific to opioid‐rich regions per se, which is corroborated by the expression of MOR throughout the brain (Mansour et al., 1988). G‐carriers displayed reduced functional connectivity between PCC/precentral gyrus and DLPFC (Figure 7), crucially involved in the fronto‐parietal network (Zanto & Gazzaley, 2013) and pain modulation (Seminowicz & Moayedi, 2017), with DLPFC previously showing reduced MOR binding potential in G‐carriers (Peciña et al., 2015). Our findings indicate alternative modulatory patterns with engagement of the fronto‐partietal network in OPRM1 AA and PCC/precentral gyrus in OPRM1 */G. Interestingly, ROIs (Schrepf et al., 2016) overlapped not only where our data displayed OPRM1 differences in neural processing (PCC/precentral gyrus) but also in functional connections (DLPFC).

An increased endogenous opioid tone in FM, suggested by elevated endogenous opioids in the CSF (Baraniuk et al., 2004), may lead to opioid‐induced hyperalgesia, shown to be associated with glial activation (Roeckel et al., 2016). Microglia activation was recently demonstrated in precuneus/PCC, S1/M1 and DLPFC, providing evidence for glial involvement in FM pathophysiology (Albrecht et al., 2019). Several of those regions were involved in differing functional patterns between OPRM1 genotypes in the current data. Some drugs presumably acting on glial cells have shown favourable effects in FM, for example, milnacipran (Clauw et al., 2008). Besides its primary mechanism, milnacipran has been shown to mitigate microglia activation in a mouse model (Shadfar et al., 2018), suggesting an effect through microglia modulation. A study in FM subjects showed that the degree of antinociceptive effect of milnacipran correlated with pain‐related BOLD signal in PCC (Jensen et al., 2014). Notably, the PCC peak coordinate in this study [−2 –28 46] was very similar to the location of the positive milnacipran response [−4 –30 46], stressing the clinical role of PCC in FM‐relevant pain modulation. As the OPRM1 effect was significant in FM subjects but not in HC, we speculate that FM subjects drive this finding.

However, we cannot exclude that OPRM1 */G may confer diminished endogenous opioid tone, due to reduced expression of MOR (Bond et al., 1998). In this sense, the diminished MOR binding potential in OPRM1 G‐carriers (Peciña et al., 2015) is inconclusive, as it could result from increased binding of endogenous ligands, therefore, preventing PET ligand binding and/or reduced MOR expression (Loggia, 2018). Arguably, the effect of OPRM1 may be due to reduced MOR availability in FM (Harris et al., 2007) caused by lower expression of opioid receptors in response to long‐term exposure to high levels of endogenous opioids. The latter would be in accordance with reports of OPRM1 */G preventing upregulation of MOR by decreasing OPRM1 mRNA expression following chronic opioid exposure in opioid addicts (Oertel et al., 2012). The effect of OPRM1 */G would then be more pronounced in FM subjects than HC, which is suggested in our data.

Decreased resting state connectivity in FM has been found between pain‐relevant and sensorimotor areas (Flodin et al., 2014). FM subjects also showed decreased coupling between supramarginal gyrus and S1/M1, brain regions partly displaying reduced connectivity with PCC in G‐allele carriers (Figure 7). In HC, prefrontal cortex and PCC/precuneus increased coupling during painful disruptions, suggesting alternative connectivity patterns between DMN and pain‐relevant regions during evoked pain (Mantini et al., 2009). We found that OPRM1 G‐carriers showed decreased connectivity between PCC/precentral gyrus and inferior parietal lobe, considered a hub for integrating multisensory information (Seghier, 2013) and DMN (Davey et al., 2016). Here, increased PCC activation in G‐carriers may indicate failed appropriate deactivation during painful stimulation, where attentional focus is presumably external. Increased connectivity in AA homozygotes may indicate deactivation of the DMN and presumably activation of the fronto‐parietal network (Leech & Sharp, 2014). One could argue that reduced control over pain‐relevant pathways in FM during rest may be complemented by additional modulations in connectivity through genetic dispositions independent of disease.

Functional data did not reveal a significant group‐by‐genotype interaction, indicating similar effects of OPRM1 in FM subjects and HC. As no correlation between PCC/precentral gyrus activation and clinical measures was found, no FM‐specific effects of OPRM1 were identified. With respect to perception, higher catastrophizing scores were linked to higher experimental pain ratings in FM subjects, regardless of genotype. Additionally, no differences in pain‐evoked brain activation between FM and HC were found with perceived pain intensity adjusted between groups, which is in line with previous reports (López‐Solà et al., 2017). However, the interaction analysis may have been insufficiently powered and differential OPRM1 effects between groups cannot be ruled out, in fact, we suggest that the OPRM1 effects were driven by the FM group. Further research is needed to investigate whether cerebral OPRM1 differences are specific to FM.

Importantly, a combination of the OPRM1 G‐allele and other factors may, nonetheless, play a role in chronic pain. G‐carriers in clinical pain cohorts have been associated with less preferential characteristics (Menon et al., 2012; Tan et al., 2009; Wei et al., 2017). However, contrasting results (Ballina et al., 2013; Linnstaedt et al., 2015) emphasize the need for more research on the role of OPRM1 in acute and chronic pain.

We did not observe OPRM1 differences in pain sensitivity or pain ratings, which is in line with studies in FM and HC (Peciña et al., 2015; Solak et al., 2014; Tour et al., 2017), however, there have been mixed reports in clinical cohorts (Menon et al., 2012) and HC (Fillingim et al., 2005). Similar to our results, no differences in other FM characteristics, for example, depression, have been observed (Solak et al., 2014; Tour et al., 2017). Given the lacking OPRM1 effect on behavioural/physiological measures, we cannot conclude that the differences in neural processing are accompanied by perceptual or clinically relevant discrepancies. We, thus, suggest that differing cerebral modulatory processes may lead to similar behavioural/perceptual outcome.

We acknowledge some caveats associated with this study. First, due to the genotype frequency in OPRM1 the absolute number of G‐allele carriers was smaller than homozygous AA, resulting in uneven subgroup sizes. Thus, some analyses may have been insufficiently powered, which limits the interpretability, particularly absent interaction effects between genotype, group and pressure level. We cannot conclude that the OPRM1 effect is specific to a certain pain intensity. Importantly, we emphasize that the observed OPRM1 differences are in need of replication in a larger sample. Second, due to lacking an affective pain measure, the current data cannot corroborate a reported association between the OPRM1 G‐allele and affective regulation (Finan et al., 2010). Finally, as FM is predominant in women, we included only female participants. Our results may, therefore, not be applicable to males.

To conclude, this study provides further evidence for a functional role of the OPRM1 polymorphism in the neural processing of evoked pain. Specifically, G‐allele carriers showed increased activation in PCC/precentral gyrus and decreased functional connectivity with the fronto‐parietal network, suggesting alternative pain modulatory mechanisms between OPRM1 variants. Finally, we speculate that the OPRM1 effect may be driven by FM subjects.

## CONFLICT OF INTEREST

There are no conflicts of interest to declare.

## AUTHORS' CONTRIBUTIONS

E.K. and K.B.J. conceptualized and designed the study. E.K. acquired funding. E.K. and M.S. provided resources. D.K. screened participants. A.S. and J.T. collected the data. M.S. provided expertise on genotyping. I.E. analysed the data and prepared the original draft. I.E., E.K. and A.S. interpreted the data. All authors discussed the results and commented on the manuscript.

## Supporting information

Figure S1

Table S1

Table S2

Table S3

Table S4
