# Supplementary figures and images for: Polymorphisms of the μ‐opioid receptor gene influence cerebral pain processing in fibromyalgia
Source: Eur J Pain. 2020 Nov 2;25(2):398–414. doi: 10.1002/ejp.1680 (PMC7821103; doi:10.1002/ejp.1680)

**a**

VAS rating

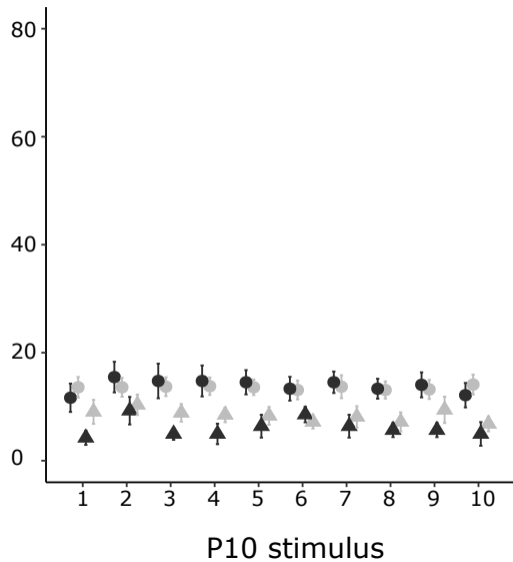**b**

VAS rating

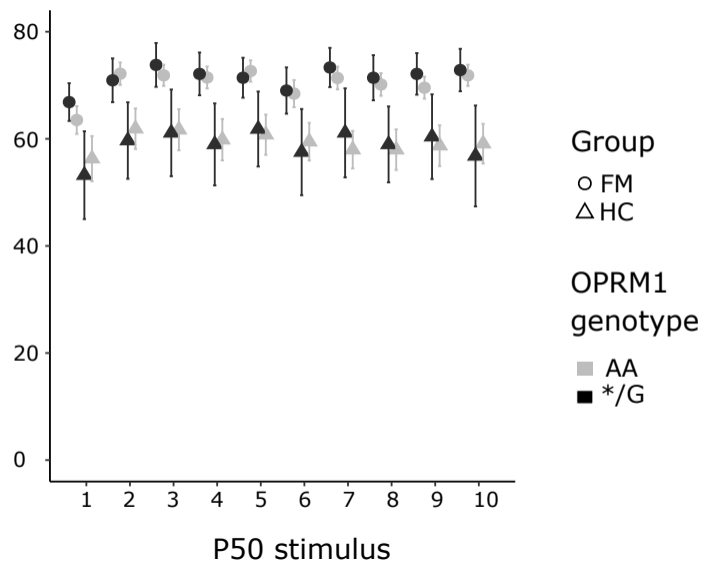

Supplement: Supplementary file 1 — Figure S1 [file EJP-25-398-s001.pdf]
