# Supplementary material for: Polymorphisms of the μ‐opioid receptor gene influence cerebral pain processing in fibromyalgia
Source: Eur J Pain. 2020 Nov 2;25(2):398–414. doi: 10.1002/ejp.1680 (PMC7821103; doi:10.1002/ejp.1680)
Supplement: Supplementary file 2 — Table S1 [file EJP-25-398-s002.docx]

|  | AA genotpye (n=77) | | */G genotype (n=28) | |
| --- | --- | --- | --- | --- |
|  | **FM**  (n=50; ~70% of FM) | **HC**  (n=27; ~79% of HC) | **FM**  (n=21; ~30% of FM) | **HC**  (n=7; ~21% of HC) |
| *Mean age in years (SD)* | 47.1 (±7.8) | 47.7 (±7.9) | 48.9 (±7.7) | 48.6 (±8.3) |
| *Median pressure in mmHg (mean, SD)* | P10: 62 (73, ±40)  P50: 169 (192, ±73) | P10: 130 (136, ±32)  P50: 302 (295, ±64) | P10: 65 (68, ±28)  P50: 204 (201, ±89) | P10: 100 (105, ±41)  P50: 249 (263, ±67) |
| *Mean PPTs in kPa (SD)* | 156 (±58.8) | 333 (±113) | 156 (±74.1) | 289 (±92.5) |
| *Mean PCS scores (SD)* | 17.4 (±10.6) | 4 (±5.7) | 17.4 (±9.4) | 7.71 (±12.2) |
| *Mean BDI scores (SD)* | 15.3 (±7.9) | 0.3 (±0.7) | 14.1 (±6.5) | 0.3 (±0.49) |
| *Mean STAI-S scores (SD)* | 42.6 (±11.9) | 28.9 (±6.8) | 43.5 (±11.8) | 32.9 (±10.3) |
| *Mean SF-36 bodily pain scores (SD)* | 30.8 (±14.1) | 90.6 (±11.2) | 34.6 (±13.8) | 86.1 (±17.4) |
| *Mean FIQ scores (SD)* | 63 (±15.7) | - | 60.1 (±17.5) | *-* |
| *Mean pain duration in months (SD, min, max)* | 190 (±115, 24, 492) | - | 196 (±86.4, 84, 408) | - |

**Table S1**.
