# Supplementary material for: Polymorphisms of the μ‐opioid receptor gene influence cerebral pain processing in fibromyalgia
Source: Eur J Pain. 2020 Nov 2;25(2):398–414. doi: 10.1002/ejp.1680 (PMC7821103; doi:10.1002/ejp.1680)
Supplement: Supplementary file 3 — Table S2 [file EJP-25-398-s003.docx]

| **Effect** | **β** | **Lower CI** | **Upper CI** | ***SE*** | ***t-*value** | ***p*-value** |
| --- | --- | --- | --- | --- | --- | --- |
| *Group* | -8.361 | -14.709 | -2.013 | 3.199 | -2.614 | 0.01* |
| *OPRM1 genotype* | 1.521 | -6.057 | 9.01 | 3.819 | 0.398 | 0.69 |
| *Pressure level* | -51.335 | -53.898 | -48.773 | 1.306 | -39.294 | <0.001* |
| *Time* | 0.139 | -0.089 | 0.367 | 0.117 | 1.193 | 0.233 |
| *PPT* | -0.003 | -0.026 | 0.02 | 0.012 | -0.236 | 0.814 |
| *OPRM1*Group* | -0.849 | -9.327 | 7.628 | 4.272 | -0.199 | 0.843 |
| *OPRM1*Pressure level* | -3.02 | -7.151 | 1.111 | 2.106 | -1.434 | 0.152 |
| *OPRM1*Time* | -0.029 | -0.44 | 0.382 | 0.21 | -0.14 | 0.891 |

**Table S2**.
