# Supplementary material for: Polymorphisms of the μ‐opioid receptor gene influence cerebral pain processing in fibromyalgia
Source: Eur J Pain. 2020 Nov 2;25(2):398–414. doi: 10.1002/ejp.1680 (PMC7821103; doi:10.1002/ejp.1680)
Supplement: Supplementary file 4 — Table S3 [file EJP-25-398-s004.docx]

**Table S3.**

| **Region/s** | **Peak coordinate** | | | **t** | **Cluster size (k)** | **p-value** |
| --- | --- | --- | --- | --- | --- | --- |
|  | **x** | **y** | **z** |  |  |  |
| ***FM P50*** |  |  |  |  |  |  |
| R Parietal Operculum  R Central Operculum  R Insula | 46  48  34 | -30  0  -20 | 20  4  12 | 17.24  15.77  15.52 | 3975 | <0.001 |
| L Parietal Operculum  L Planum Polare /  L Precentral Gyrus  L Insula | -52  -56  -36 | -32  4  2 | 20  0  8 | 12.57 | 1801 | <0.001 |
| R JLC / R Precentral Gyrus  R Postcentral Gyrus  R Postcentral Gyrus | 6  16  6 | -12  -40  -38 | 64  68  62 | 12.01  11.88  10.02 | 1267 | <0.001 |
| R ACC / R Paracingulate Gyrus  R ACC / R JLC | 4  2 | 14  -8 | 40  46 | 8.93 | 745 | <0.001 |
| R Putamen  R Amygdala | 18  22 | 14  4 | -6  -14 | 6.67  5.50 | 33 | 0.002 |
| R Frontal Pole | 46 | 40 | 2 | 6.41 | 28 | 0.003 |
| L Insula | -38 | -18 | -2 | 5.32 | 6 | 0.018 |
|  |  |  |  |  |  |  |
| ***FM P10*** |  |  |  |  |  |  |
| R Parietal Operculum  R Parietal Operculum / R Supramarginal Gyrus  R Insula / R Frontal Operculum | 48  58  34 | -28  -22  24 | 20  22  4 | 17.80  15.08  14.23 | 4748 | <0.001 |
| L Parietal Operculum  L Central Operculum  L Insula | -50  -42  -32 | -34  -2  18 | 20  10  6 | 13.47  12.59  12.31 | 3767 | <0.001 |
| R Paracingulate Gyrus  L Paracingulate Gyrus  L Paracingulate Gyrus | 4  -4  -6 | 22  12  20 | 42  48  40 | 13.03  10.18  10.07 | 1149 | <0.001 |
| R Postcentral Gyrus | 16 | -38 | 70 | 12.55 | 357 | <0.001 |
| R Frontal Pole  R Frontal Pole / R Middle Frontal Gyrus | 46  44 | 46  38 | 4  20 | 9.83  8.46 | 531 | <0.001 |
| R Putamen / R Caudate | 16 | 16 | -4 | 9.45 | 119 | <0.001 |
| L Putamen | -18 | 10 | -6 | 6.64 | 51 | <0.001 |
| L Frontal Pole | -44 | 40 | 8 | 6.29 | 51 | <0.001 |
| R JLC / R Precentral Gyrus | 6 | -10 | 64 | 5.61 | 37 | <0.001 |
|  |  |  |  |  |  |  |
| ***HC P50*** |  |  |  |  |  |  |
| R Postcentral Gyrus  R Postcentral Gyrus  R Precentral Gyrus | 14  10  6 | -36  -40  -24 | 72  64  64 | 10.74  9.43  9.28 | 486 | <0.001 |
| R Insula  R Insula  R Central Operculum | 36  34  56 | 14  6  6 | 8  8  4 | 9.68  8.65  8.18 | 301 | <0.001 |
| R Parietal Operculum  R Parietal Operculum /  R Supramarginal Gyrus | 44  58 | -28  -24 | 20  22 | 9.46  9.04 | 342 | <0.001 |
| L Central Operculum / L Insula | -36 | 2 | 12 | 8.90 | 85 | <0.001 |
| R Insula / R Parietal Operculum | 32 | -20 | 14 | 6.56 | 16 | 0.003 |
| R Frontal Operculum / R Temporal Pole | 48 | 14 | -4 | 6.21 | 13 | 0.004 |
| R ACC | 8 | -6 | 42 | 6.18 | 6 | 0.011 |
| R Insula | 38 | -10 | -4 | 6.09 | 16 | 0.003 |
|  |  |  |  |  |  |  |
| ***HC P10*** |  |  |  |  |  |  |
| R Parietal Operculum / R Supramarginal Gyrus  R Parietal Operculum  R Insula | 60  46  34 | -22  -28  -18 | 24  20  10 | 12.62  11.16  8.65 | 778 | <0.001 |
| L Postcentral Gyrus / L Supramarginal Gyrus  L Parietal Operculum | -62  -46 | -22  -28 | 22  20 | 9.62  6.91 | 205 | <0.001 |
| R Insula  R Precentral Gyrus / R Central Operculum  R Insula | 34  58  36 | 16  6  4 | 6  4  6 | 9.46  8.11  8.05 | 522 | <0.001 |
| R Postcentral Gyrus  R Postcentral Gyrus | 16  10 | -38  -40 | 74  64 | 7.99  6.50 | 60 | <0.001 |
| L Central Operculum / L Insula | -42 | -2 | 10 | 7.85 | 103 | <0.001 |
| L Frontal Operculum / L Insula | -36 | 14 | 6 | 7.60 | 47 | <0.001 |
