# Supplementary material for: Polymorphisms of the μ‐opioid receptor gene influence cerebral pain processing in fibromyalgia
Source: Eur J Pain. 2020 Nov 2;25(2):398–414. doi: 10.1002/ejp.1680 (PMC7821103; doi:10.1002/ejp.1680)
Supplement: Supplementary file 5 — Table S4 [file EJP-25-398-s005.docx]

| **Table S4.**  **Region/s** | **Peak coordinate** | | | **t** | **Cluster size (k)** | **p-value** |
| --- | --- | --- | --- | --- | --- | --- |
|  | **x** | **y** | **z** |  |  |  |
| ***FM cue-anticipation (P50)*** |  |  |  |  |  |  |
| L Supramarginal Gyrus  L Parietal Operculum  L Supramarginal Gyrus | -64 -48 -62 | -30  -34  -40 | 20  22  32 | 8.41  7.64  7.38 | 593 | <0.001 |
| R Planum Polare / R Parietal Operculum  R Parietal Operculum  R Supramarginal Gyrus | 62  48  64 | -24  -30  -44 | 16  20  16 | 7.47  7.35  7.34 | 522 | <0.001 |
| R Central Operculum | 58 | 2 | 4 | 7.12 | 192 | <0.001 |
| L Occipital Lobe  L Lateral Occipital Cortex | -14  -10 | -90  -82 | 32  44 | 7.06  5.33 | 69 | <0.001 |
| L Planum Polare / L Parietal Operculum | -58 | -2 | 2 | 6.24 | 87 | <0.001 |
| L Lateral Occipital Cortex  L Middle Temporal Gyrus  L Lateral Occipital Cortex | -58  -60  -54 | -62  -50  -70 | 6  8  10 | 6.12  5.70  5.22 | 121 | <0.001 |
| R Precentral Gyrus | 52 | 0 | 50 | 5.82 | 13 | 0.008 |
| R Lateral Occipital Cortex | 56 | -64 | 8 | 5.82 | 7 | 0.015 |
| L Precuneus | -6 | -60 | 58 | 5.51 | 38 | 0.001 |
|  |  |  |  |  |  |  |
| ***FM cue-anticipation (P10)*** |  |  |  |  |  |  |
| R Parietal Operculum | 48 | -30 | 22 | 8.73 | 251 | <0.001 |
| L Supramarginal Gyrus /  L Parietal Operculum  L Parietal Operculum | -60  -48 | -28  -36 | 22  20 | 7.72  7.12 | 377 | <0.001 |
| R Postcentral Gyrus  R Precentral Gyrus  R Precentral Gyrus /  R Postcentral Gyrus | 6  8  12 | -40  -16  -32 | 62  68  68 | 7.60  7.53  7.26 | 491 | <0.001 |
| L Precuneus  L Lateral Occipital Cortex  L Occipital Pole | -6  -8  -10 | -80  -76  -88 | 42  52  36 | 66.68  5.42  5.11 | 38 | <0.001 |
| R Lingual Gyrus | 12 | -42 | -6 | 6.50 | 48 | <0.001 |
| L Central Operculum | -54 | -2 | 8 | 5.86 | 22 | 0.002 |
| L Postcentral Gyrus | -18 | -46 | 64 | 5.68 | 11 | 0.006 |
| L PCC / L Precentral Gyrus | -14 | -26 | 38 | 5.66 | 11 | 0.006 |
| L Lateral Occipital Cortex | -56 | -64 | 2 | 5.45 | 14 | 0.004 |
|  |  |  |  |  |  |  |
| ***HC cue-anticipation (P50)*** |  |  |  |  |  |  |
| R Postcentral Gyrus  R Precentral Gyrus  R Precentral Gyrus | 6  8  4 | -36  -28  -22 | 60  58  62 | 9.11  9.01  8.73 | 1491 | <0.001 |
| L Postcentral Gyrus /  L Superior Parietal Lobe  L Postcentral Gyrus | -20  -16 | -46  -42 | 66  58 | 8.93  8.60 | 297 | <0.001 |
| L Parietal Operculum  L Parietal Operculum | -58  -50 | -32  -36 | 20  22 | 7.67  7.02 | 97 | <0.001 |
| R Parietal Operculum | 54 | -28 | 20 | 6.96 | 38 | <0.001 |
| L ACC | -6 | -4 | 42 | 6.90 | 36 | <0.001 |
| R ACC | 6 | -8 | 42 | 6.37 | 18 | 0.002 |
| L Precentral Gyrus / L PCC | -14 | -32 | 40 | 6.18 | 16 | 0.003 |
| L Central Operculum | -58 | 0 | 4 | 6.14 | 10 | 0.006 |
|  |  |  |  |  |  |  |
| ***HC cue-anticipation (P10)*** |  |  |  |  |  |  |
| L Postcentral Gyrus  L Postcentral Gyrus | -20  -16 | -46  -44 | 68  60 | 8.31  6.47 | 72 | <0.001 |
| R Precentral Gyrus  R Postcentral Gyrus  R Precentral Gyrus /  R Postcentral Gyrus | 6  6  12 | -24  -38  -32 | 66  58  66 | 7.82  7.65  7.53 | 391 | <0.001 |
| R Central Operculum | 56 | 4 | 4 | 6.09 | 7 | 0.007 |
