# Supplementary material for: Polymorphisms of the μ‐opioid receptor gene influence cerebral pain processing in fibromyalgia
Source: Eur J Pain. 2020 Nov 2;25(2):398–414. doi: 10.1002/ejp.1680 (PMC7821103; doi:10.1002/ejp.1680)
Supplement: Supplementary file 6 [file EJP-25-398-s006.docx]

**Table S1**. **Characteristics of OPRM1 genotypes reported for fibromyalgia (FM) subjects and healthy controls (HC) included in fMRI analysis (n=105)**

SD = standard deviation, min = minimum, max = maximum, mmHg = millimetres of mercury, kPa = kilopascal, PCS = pain catastrophizing scale, BDI = Beck’s depression inventory, STAI-S = State-trait anxiety inventory (state), FIQ = fibromyalgia impact questionnaire

**Table S2. Linear mixed model results for predictors of pain ratings for subjects included in fMRI analysis (n=105)**

PPT = pressure pain threshold, β = beta estimate, CI = 95% confidence interval, SE = standard error, * significant at p<0.05

**Table S3. Localization of significant clusters (p < 0.05, whole brain FWE-corrected) during pressure pain stimulation matching 10/100 VAS (P10) and 50/100 VAS (P50) in fibromyalgia subjects (FM) and healthy controls (HC)**

Anatomical site, maximum *t* value, and MNI coordinates (in millimeters) of the local maxima. L = left, R = right, Parietal Operculum = S2, Postcentral Gyrus = S1, Precentral Gyrus = M1, JLC = Juxtapositional Lobule Cortex, ACC = Anterior Cingulate Cortex

**Table S4. Localization of significant clusters (p < 0.05, whole brain FWE-corrected) showing differences during cue-anticipation phase in fibromyalgia (FM) subjects and healthy controls (HC)**

Anatomical site, maximum *t* value, and MNI coordinates (in millimeters) of the local maxima. L = left, R = right, Parietal Operculum = S2, Postcentral Gyrus = S1, Precentral Gyrus = M1, PCC = Posterior Cingulate Cortex, ACC = Anterior Cingulate Cortex

**Figure S1. Pain ratings for OPRM1 genotype A (n=77) and OPRM1 G-carriers (n=28) in response to painful pressure stimuli displayed for participants in the fMRI analysis (n=105)**

Individual pain ratings were acquired using a visual analogue scale (VAS) ranging from “no pain” (0) to “worst pain imaginable” (100). There was a significant difference between groups (FM/HC) and between stimulus intensities (P10/P50) but no difference between OPRM1 genotypes (AA/*G). **(a)** Pain ratings are displayed for stimuli of 10/100 VAS (P10) and **(b)** for 50/100 VAS (P50). Error bars represent the standard error of the mean. FM = Fibromyalgia, HC = Healthy controls
